# Association of bradykinin receptor 2 (BDKRB2) variants with physical performance and muscle mass: Findings from the LACE sarcopenia trial

**DOI:** 10.1371/journal.pone.0307268

**Published:** 2024-08-02

**Authors:** Alvin Shrestha, Tufail Bashir, Marcus Achison, Simon Adamson, Asangaedem Akpan, Terry Aspray, Alison Avenell, Margaret M. Band, Louise A. Burton, Vera Cvoro, Peter T. Donnan, Gordon W. Duncan, Jacob George, Adam L. Gordon, Celia L. Gregson, Adrian Hapca, Cheryl Hume, Thomas A. Jackson, Simon Kerr, Alixe Kilgour, Tahir Masud, Andrew McKenzie, Emma McKenzie, Harnish Patel, Kristina Pilvinyte, Helen C. Roberts, Avan A. Sayer, Christos Rossios, Karen T. Smith, Roy L. Soiza, Claire J. Steves, Allan D. Struthers, Divya Tiwari, Julie Whitney, Miles D. Witham, Paul R. Kemp

**Affiliations:** 1 Cardiovascular and Respiratory Interface Section, National Heart and Lung Institute, Imperial College London, South Kensington Campus, London, United Kingdom; 2 Cutrale Perioperative and Ageing Group, Department of Bioengineering, Imperial College London, London, United Kingdom; 3 Tayside Clinical Trials Unit (TCTU), Tayside Medical Science Centre (TASC), Ninewells Hospital & Medical School, University of Dundee, Dundee, United Kingdom; 4 Liverpool University Hospitals NHS FT Trust, Clinical Research Network Northwest Coast, University of Liverpool, Liverpool, United Kingdom; 5 AGE Research Group, NIHR Newcastle Biomedical Research Centre, Cumbria Northumberland Tyne and Wear NHS Foundation Trust and Newcastle upon Tyne Hospitals NHS Trust, Translational Clinical Research Institute, Newcastle University, Newcastle upon Tyne, United Kingdom; 6 Health Services Research Unit, University of Aberdeen, Aberdeen, United Kingdom; 7 Medicine for the Elderly, NHS Tayside, Dundee, United Kingdom; 8 Ageing and Health, University of Dundee, Dundee, United Kingdom; 9 Victoria Hospital, Kirkcaldy, United Kingdom; 10 Centre for Clinical Brain Sciences, University of Edinburgh, Edinburgh, United Kingdom; 11 Division of Population Health and Genomics, School of Medicine, University of Dundee, Dundee, United Kingdom; 12 Medicine for the Elderly, NHS Lothian, Edinburgh, United Kingdom; 13 Division of Molecular & Clinical Medicine, University of Dundee Medical School, Ninewells Hospital, Dundee, United Kingdom; 14 Unit of Injury, Inflammation and Recovery, School of Medicine, University of Nottingham, Nottingham United Kingdom; 15 NIHR Nottingham Biomedical Research Centre, Department of Medicine for the Elderly, University Hospitals of Derby and Burton NHS Foundation Trust, Derby, United Kingdom; 16 Musculoskeletal Research Unit, Bristol Medical School, University of Bristol, Bristol, United Kingdom; 17 Older Person’s Unit, Royal United Hospital NHS Foundation Trust Bath, Bath, United Kingdom; 18 Institute of Inflammation and Ageing, University of Birmingham, Birmingham, United Kingdom; 19 Department of Older People’s Medicine, Newcastle upon Tyne Hospitals NHS Foundation Trust, Newcastle upon Tyne, United Kingdom; 20 Ageing and Health Research Group, Usher Institute, University of Edinburgh, Edinburgh, United Kingdom; 21 Clinical Gerontology Research Unit, Nottingham University Hospitals NHS Trust, City Hospital Campus, Nottingham, United Kingdom; 22 NIHR Biomedical Research Centre, University of Southampton and University Hospital Southampton NHSFT, Southampton, Hampshire, United Kingdom; 23 Academic Geriatric Medicine, Mailpoint 807 Southampton General Hospital, University of Southampton, Southampton, United Kingdom; 24 Ageing & Clinical Experimental Research (ACER) Group, University of Aberdeen, Aberdeen, United Kingdom; 25 Department of Twin Research and Genetic Epidemiology, King’s College London & Department of Clinical Gerontology, King’s College Hospital, London, United Kingdom; 26 Bournemouth University and Royal Bournemouth Hospital, Bournemouth, United Kingdom; 27 School of Population Health & Environmental Sciences, King’s College London and King’s College Hospital, London, United Kingdom; University Hospital of Padova, ITALY

## Abstract

**Introduction:**

Understanding genetic contributors to sarcopenia (age-related loss of muscle strength and mass) is key to finding effective therapies. Variants of the bradykinin receptor 2 (BDKRB2) have been linked to athletic and muscle performance. The rs1799722–9 and rs5810761 T alleles have been shown to be overrepresented in endurance athletes, possibly due to increased transcriptional rates of the receptor. These variants have been rarely studied in older people or people with sarcopenia.

**Methods:**

We performed a post hoc sub-study of the Leucine and ACE (LACE) inhibitor trial, which enrolled 145 participants aged ≥70 years with low grip strength and low gait speed. Participants’ blood samples were genotyped for rs179972 using TaqMan and rs5810761 by amplification through Hotstar Taq. Genotypes were compared with outcomes of physical performance and body composition measures.

**Results:**

Data from 136 individuals were included in the analysis. For rs1799722 the genotype frequency (TT: 17, CC: 48, CT: 71) remained in Hardy-Weinberg Equilibrium (HWE p = 0.248). There was no difference between the genotypes for six-Minute Walk Distance (6MWD) or Short Physical Performance Battery (SPPB). Men with the TT genotype had a significantly greater 6MWD than other genotypes (TT 400m vs CT 310m vs CC 314m, p = 0.027), and greater leg muscle mass (TT 17.59kg vs CT 15.04kg vs CC 15.65kg, p = 0.007). For rs5810761, the genotype frequency (-9-9: 31, +9+9: 43, -9+9: 60) remained in HWE (p = 0.269). The +9+9 genotype was associated with a significant change in SPPB score at 12 months (-9-9 0 vs -9+9 0 vs +9+9–1, p<0.001), suggesting an improvement. In men, the -9-9 genotype was associated with lower arm fat (-9-9 2.39kg vs -9+9 2.72kg vs +9+9 2.76kg, p = 0.019).

**Conclusion:**

In men, the rs1799722 TT genotype was associated with longer 6MWD and greater leg muscle mass, while the rs5810761 -9-9 genotype was associated with lower arm fat mass.

## Introduction

Sarcopenia is the age-related decline in muscle strength and muscle mass. It is associated with adverse outcomes such as falls, fractures, reduced quality of life and increased mortality [1]. The loss of muscle mass occurs as a result of an imbalance in protein synthesis and protein degradation with reduced synthesis compared to degradation leading to net loss of muscle protein. Whilst this loss of muscle mass can contribute to the loss of muscle strength and physical performance, changes in performance may also reflect changes in energy provision (e.g. mitochondrial dysfunction or blood flow) and/or changes in muscle fibre-type [2]. As a result, factors that contribute to the control of muscle mass, energy metabolism and fibre type may contribute to the development of sarcopenia [1].

One system implicated in skeletal muscle loss is the renin angiotensinogen system (RAS) [3]. Classically, this pathway involves angiotensinogen converting enzyme (ACE) converting angiotensin I to angiotensin II, which acts on angiotensin II type 1 receptors (AT1R). Angiotensin II has been shown to promote muscle loss in animal models [4] and to increase the expression of MuRF1, one of the major ubiquitin ligases that control muscle protein turnover [5]. One further consequence of AT1R stimulation is the production of reactive oxygen species (ROS) which may also contribute to muscle wasting [3].

Variations in ACE synthesis as a consequence of polymorphism in the ACE gene have also been implicated in physical performance. For example, an insertion(I)/deletion (D) polymorphism is thought to account for 47% of variation in ACE expression and thereby activity, with the D allele associating with greater ACE activity and therefore higher angiotensin II [6]. The I allele is associated with better sporting performance among endurance athletes, while the D allele is associated with performance in explosive events such as sprints [7]. As explosive events tend to be associated with greater muscle mass and angiotensin II promotes muscle loss, this association is counterintuitive. However, the explanation may lie in the effects of ACE polymorphism on the types of fibre present. Skeletal muscle is comprised of two basic muscle fibre types–slow-type I, used in endurance activities, and fast-type II used for explosive power events such as sprinting. The ACE I allele has been associated with a higher proportion of type I muscle fibres which may explain its link with enhanced endurance performance [8]. We have also recently shown that the I allele is under-represented in sarcopenic men and that sarcopenic men with the I allele had higher leg strength than DD individuals suggesting a role for the RAS in sarcopenia, at least in men [9].

The RAS is also closely linked to the kallikrein system, whereby ACE breaks down bradykinin into inactive peptides. Increased bradykinin activity may contribute to the positive effect of reduced ACE activity on performance. Locally in muscle, bradykinin is thought to promote glucose uptake and alter muscle blood flow through the bradykinin receptor 2 (BDKRB2) which may in turn impact on muscle performance [10]. Thus, it is possible that the BDKRB2 could contribute to muscle performance directly through bradykinin, which in turn is partly regulated by ACE activity.

Consistent with a role for BDKRB2 in muscle physiology, several studies have indicated that genetic variants of BDKRB2 associate with athletic performance. The rare -9-9 genotype of rs5810761 has been associated with increased skeletal muscle contraction efficiency in healthy volunteers [10] and is also overrepresented in Iron-man athletes [11]. Furthermore, among elite athletes, -9 allele frequency has been found to be higher with increasing distance running events (≤200m, 400-3000m and ≥5000m events), suggesting the -9 allele is associated with endurance running [10]. The -9 allele of this polymorphism may be associated with higher expression of BDKRB2 [12]. Another variant, the single nucleotide polymorphism rs1799722, also associates with athletic performance, with the minor T allele overrepresented in marathon runners [13]. The GTEx database demonstrates this T allele to be associated with higher BDKRB2 expression [14].

The effect of the BDKRB2 polymorphisms on specific muscle outcomes or within disease has rarely been studied. Hopkinson et al. did find in those with COPD, the -9 allele was an independent predictor for increased fat-free muscle index (FFMI) [15]. However, these polymorphisms have not been studied in older people with sarcopenia. If BDKRB2 polymorphism alleles -9 and T do improve physical performance or muscle attributes (e.g. FFMI) through increased BDKRB2 activity, we may expect underrepresentation of the variants within a sarcopenic population, and also similar differences in improved muscle performance in these genotype groups.

Using samples and observations from the Leucine or Angiotensin Converting Enzyme inhibitors for sarcopenia (LACE) trial, we sought to investigate genotypes of rs1799722 and rs5810761, firstly to find if their frequencies in a sarcopenic population would deviate from known previous control or population frequencies, and secondly, if the genotypes would associate with measures of muscle performance.

## Methods

### Participant selection

The LACE study was a randomised, controlled trial investigating the effects of the ACE-inhibitor Perindopril and/or leucine on measures of physical performance and muscle mass in older people with sarcopenia, compared with placebo. LACE recruited 145 participants between April 2016 and December 2019, and the population was >99% white [16]. Eligibility criteria included ≥70 years of age and above and meeting the criteria for sarcopenia, which were low skeletal muscle mass (from bioimpedance), with low gait speed and low handgrip strength. Written informed consent was obtained from all participants at enrolment. The trial was approved by the East of Scotland Ethics Committee (ref 14/ES/1099) and the UK Medicines and Healthcare Regulatory Authority (EudraCT number 2014-003455-61; Clinical Trial Authorisation number 36888/0001/001-0001). The study is registered online (www.isrctn.com, ISRCTN90094835) and was performed in accordance with the 1964 Declaration of Helsinki. The trial protocol has been published [17]. The study did not find any significant effect of ACE inhibitors on the primary outcome of change in short physical performance battery (SPPB) score or secondary outcomes of muscle performance (muscle mass, grip strength, quadriceps maximal voluntary contraction [QMCV], six-minute walk distance [6MWD], gait speed, chair-stand time); the results have been published previously [16].

We used physiological data and baseline blood samples from the LACE trial to perform this sub-study on genetic variants of BDKRB2.

### Genotyping

DNA was extracted from whole blood as described previously [18] and the samples were genotyped for rs179972 using TaqMan ABI 7500 fast thermocycler. Alleles were determined by visualisation of amplification plots. rs5810761 was genotyped by amplification through Hotstar Taq DNA polymerase kit. 4% agarose gel electrophoresis was then performed using SYBRsafe staining to identify genotype by determining product sizes on GelDoc imager. 136 participants were successfully genotyped for rs1799722, while 134 participants were genotyped for rs5810761. The remaining samples had inadequate DNA or were missing.

### Physiological measurements

The SPPB score is a tool for measuring lower extremity function, involving balance and strength, with higher scores indicating better performance. Other measurements obtained were gait speed, which required participants to walk at normal pace for 4 metres, and the chair-stand test, which is time taken to perform 5 ‘sit to stand’ chair rises; both tests form part of the SPPB. Muscle mass was measured by dual energy x-ray absorptiometry (DXA) scan. Handgrip strength was determined by a hand-held Jamar dynamometer using the Southampton protocol [19]. QMVC was measured through isometric voluntary knee extension as described previously [18]. Endurance was measured with a 6MWD. All the aforementioned tests were measured at baseline, 6 and 12 months.

### Statistical analyses

Statistical analyses were performed using SPSS version 29. The dataset used in the analyses is available in S1 File. Distribution of each variant’s genotype was compared to a known population (control) using Chi-squared test to determine if they remained in Hardy-Weinberg equilibrium. The control group distribution of alleles for rs1799722 was taken from the European group from Allele Frequency Aggregator (n = 81,468), with minor allele frequency of 0.43 [20]. The control group for rs5810761 was obtained from control groups of a previous study that involved a healthy Caucasian population (as this was not available from Allele Frequency Aggregator) recruited in England, with a minor allele frequency of 0.5 [15].

Continuous variables were assessed for normal distribution using histogram and Q-Q plots. Differences in continuous variables between different genotypes were calculated using one-way analysis of variance (ANOVA) if normally distributed, or Kruskal-Wallis test if not. Post-hoc pairwise comparisons were then conducted with Tukey’s or Dunn’s comparison respectively. Such analyses were performed for all participants in parameters that were independent of differences in sex, such as improvement in 6MWD at 12 months) and improvement in grip strength at 12 months. Differences in SPPB, baseline grip strength, baseline 6MWD, QMVC, muscle and fat mass were also analysed separately for men and women given the known differences in baseline normative values. No correction was made for multiple statistical testing.

## Results

The baseline demographics of the cohort is shown in Table 1.

**Table 1:** Baseline characteristics.

	Female	Male
N	72 (53%)	64 (47%)
Age, years	76.5 (74–82)	77.5 (74–84)
Weight, kg	63.1 (57.3–71.1)	80.5 (73.5–89.6)
Height, cm	157 (± 3.0)	171 (± 3.2)
BMI, kg/m^2^	26.1 (±1.81)	27.7 (± 1.92)
Appendicular lean mass, kg*	5.78 (5.51–6.02)	7.29 (6.86–7.67)
SPPB baseline	7 (6–9)	8 (5.25–9)
Grip strength baseline, kg	13.5 (± 1.7)	22.9 (± 2.9)
QMVC baseline, kg	9.7 (6.75–12.7)	15.5 (11.63–20.75)

BMI body mass index, SPPB short physical performance battery, QMVC quadricep maximal voluntary contraction

*from bioimpedance measures

Normally distributed data is presented as mean (± SD) and non-normally data as median (interquartile range).

### BDKRB2 genotypes

The European control group (n = 81,468) has an allele frequency of C 57% and T 43% [20] for rs1799722 and the control population for rs5810761 had an allele frequency of +9 50% and -9 50% [15]. Comparison of the genotype frequencies in the LACE cohort for these two alleles did not show any difference from the control values and both alleles were in Hardy-Weinberg equilibrium, as demonstrated in Table 2.

**Table 2:** Distribution of genotype in study and population.

**rs1799722**				
By genotype:				
	Minor TT	Heterozygous CT	Major CC	P value
Observed	17	71	48	0.248
Expected	24	67	45	
By allele:				
	T	C		P value
Observed	105	167		0.143
Expected	117	155		
**rs5810761**				
By genotype:				
	-9-9	-9+9	+9+9	P value
Observed	31	60	43	0.269
Expected	35	64	35	
By allele:				
	-9	+9		P value
Observed	122	146		0.1426
Expected	134	134		

### rs1799722 variant

Comparison of the physical performance outcomes least likely to show gender differences (grip strength improvement at 12 months, 6MWD, 6MWD improvement at 12 months, SPPB score, SPPB score improvement at 12 months, chair-stand time, gait time) did not show any effects of genotype (Table 3).

**Table 3:** Association of rs1799722 genotypes on muscle function and body composition.

All participants: n = 136								
	TT			CT			CC			P value	
6MWD, m		350 (278–410)			310 (241–370)			304 (206–376)			0.227	
6MWD improvement 12m, m		-1 (-41-25)			12 (-13-45)			18 (-19.5–52.5)			0.465	
SPPB		8.0 (6.5–9.0)			7.0 (5.0–9.0)			7.0 (6.0–9.0)			0.294	
SPPB improvement 12m		0 (-1-1)			1 (0–2)			0 (-1-1)			0.163	
Chair-stand time, sec		26.3 (18.0–27.9)			20.4 (15.1–29.5)			22.6 (15.1–29.5)			0.439	
Gait speed, m/s		0.80 (±0.21)			0.76 (±0.24)			0.75 (±0.23)			0.750	
Grip strength improvement 12m, kg		-0.70 (-2.60–1.30)			1.05 (-3.77–3.60)			-0.30(-1.68–1.38))			0.191	
By gender:												
	Male n = 64						Female n = 72					
	TT		CT	CC		P	TT		CT	CC		P
Grip strength, kg	24.9 (20.8–31.0)		21.7 (18.9–25.7)	23.6 (20.6–25.8)		0.294	14.2 (11.9–18.2)		12.9 (10.1–16.3)	14.7 (10.7–16.8)		0.565
6MWD, m	400 (362–441)		310 (240–399)	314 (172–386)		0.027	278 (228–343)		297 (249–369)	300 (267–371)		0.778
QMVC, kg	16.0 (12.3–22.8)		15.8 (12.7–21.2)	14.1 (8.7–18.4)		0.349	8.1 (6.6–10.8)		10.3 (6.7–13.5)	9.9 (6.7–13.4)		0.583
Arm fat mass, kg	2.65 (1.95–3.01)		2.98 (2.35–3.25)	2.51 (1.94–3.56)		0.551	3.17 (2.57–4.23)		2.94 (1.93–4.28)	3.02 (2.43–3.53)		0.722
Leg fat mass, kg	7.22 (6.61–8.94)		6.51 (5.37–7.79)	6.40 (5.16–9.01)		0.403	8.45 (6.33–13.67)		9.09 (7.48–11.81)	9.96 (8.04–12.27)		0.848
Arm muscle mass, kg	6.27 (5.89–6.91)		5.65 (5.11–6.48)	5.74 (5.12–6.51)		0.220	3.37 (2.95–3.76)		3.60 (2.96–3.87)	3.24 (2.94–3.76)		0.455
Leg muscle mass, kg	17.6 (15.9–18.8)		15.0 (13.3–16.8)	15.7 (14.7–17.2)		0.007	9.70 (8.84–11.3)		10.9 (9.59–11.9)	10.4 (9.29–11.8)		0.265

6MWD six-minute walk distance; QMVC quadricep maximal voluntary contraction

**Fig 1:**
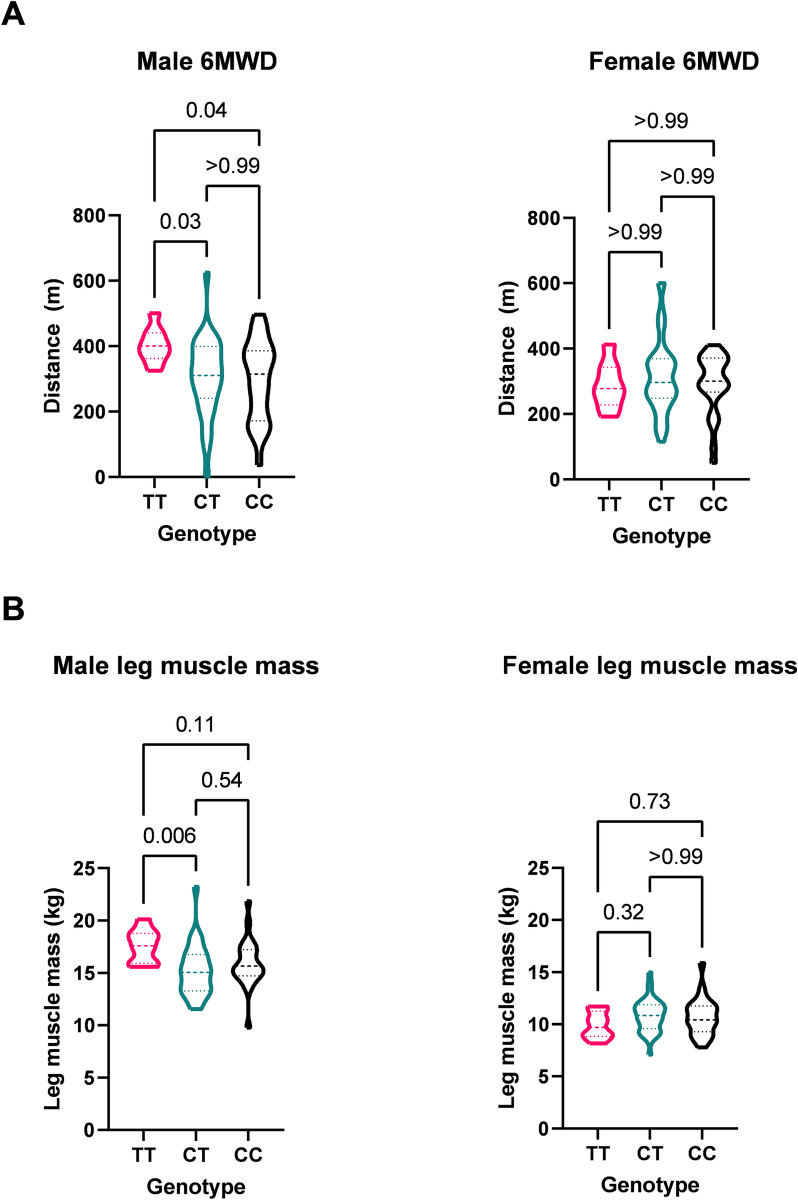
Associations of rs1799722 genotype on (A) six-minute walk distance (6MWD) and (B) leg muscle mass in men and women. Differences across the genotypes were calculated by Kruskall-Walis Test with post hoc analysis of differences between genotypes corrected using Bonferoni adjustment.

However, given the differences in normative values between men and women, associations of genotype with physiological outcomes were explored in a post hoc gender-specific manner. In men, whilst handgrip strength and QMVC did not differ, 6MWD was different across the three genotypes (p = 0.027), with those with the TT genotype having significantly greater 6MWD than the other genotypes (TT vs CT p = 0.031, TT vs CC p = 0.042, Table 3 and Fig 1). Similarly, in men, there was also a difference in baseline leg muscle mass between the genotypes (p = 0.007). Post-hoc analysis showed that individuals with the TT genotype had higher leg muscle base compared to CT (TT (17.59kg vs CT 15.04kg, p = 0.006, Fig 1). However, although median leg muscle mass was higher in TT men compared to CC men, this difference did not reach statistical significance (p = 0.112). To determine whether this lack of difference was the result of the relatively small group sizes we grouped the CC and CT genotypes to determine the effect of presence of the C allele and analysed the data. In this analysis the TT group had a larger muscle mass than the CC/CT group (p = 0.005). Analysis of the females as a group alone showed no associations with any of the physiological variables.

### rs5810761 variant

**Fig 2:**
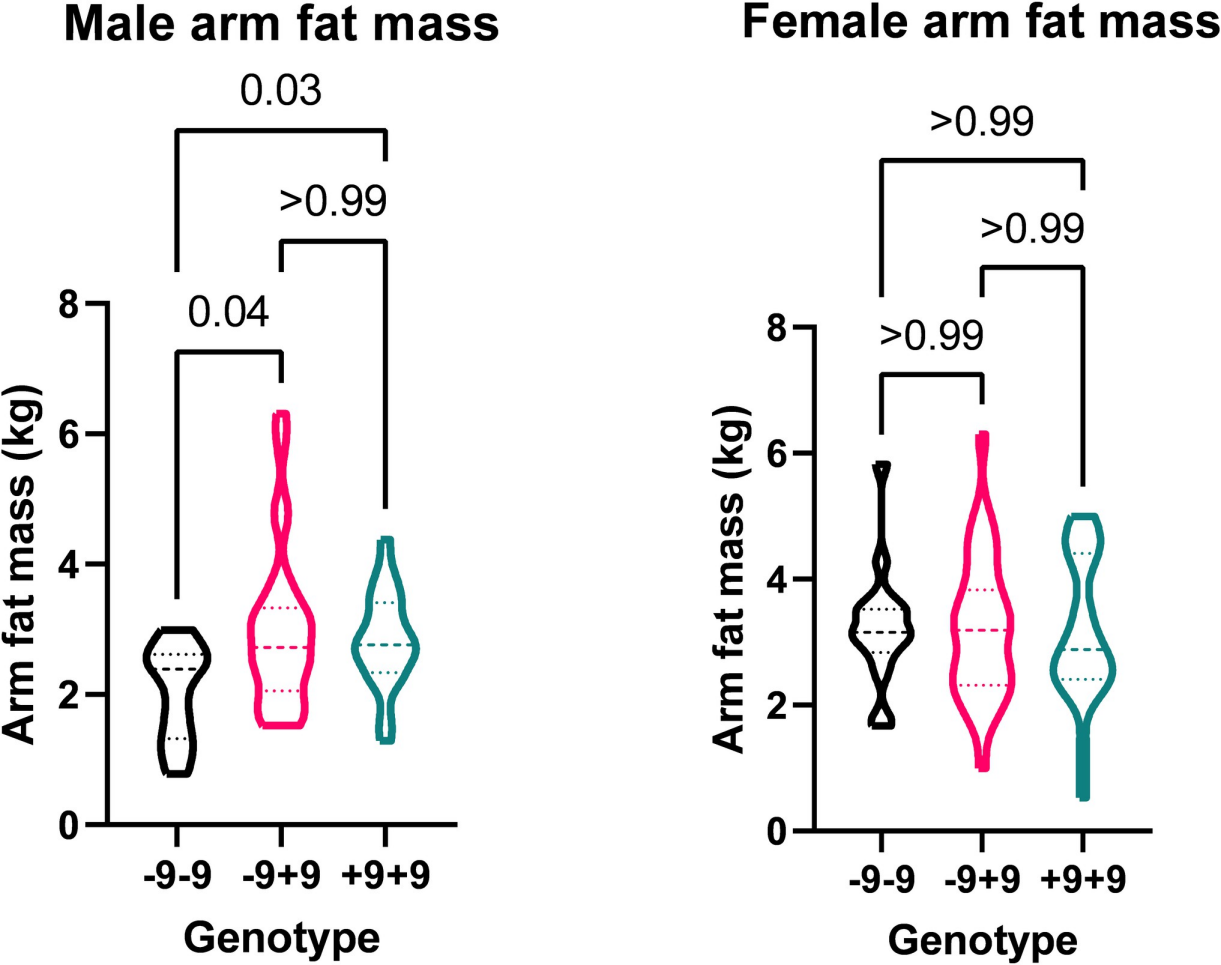
Association of rs5810761 genotype on arm fat mass in men and women. Differences across the genotypes were calculated by Kruskall-Walis Test with post hoc analysis of differences between genotypes corrected using Bonferroni adjustment.

Comparisons between genotypes for the rs5810761 were also performed as seen in Table 4. After splitting by gender, among men there was a difference in baseline arm fat between the genotypes (p = 0.019), as shown in Fig 2. The -9-9 group had lower arm fat mass of 2.39kg (1.33–2.62kg) than +9+9 2.76kg (2.33–3.41kg, p = 0.029) and -9+9 2.72kg (2.05–2.33kg, p = 0.034).

**Table 4:** Association of rs5810761 genotypes on muscle function and body composition.

All participants n = 134												
		-9-9			-9+9			+9+9			P value	
6MWD		308 (250–376)			324 (259–377)			293 (199–380)			0.506	
6MWD improvement 12m, m		23 (-21-57)			2 (-29.5–55.8)			27 (-38.0–50.0)			0.318	
SPPB		7.0 (6.0–9.0)			7.0 (6.0–9.0)			7.0 (5.0–9.0)			0.533	
SPPB improvement 12m		0.0 (1.0–1.0)			0.0 (-1.0–1.3)			1.0 (1.0–2.0)			<0.001	
Chair-stand time, sec		20.4 (15.0–26.3)			21.9 (14.9–27.9)			23.3 (19.2–29.6)			0.192	
Gait speed, m/s		5.0 (4.2–6.1)			5.3 (4.5–6.7)			5.1 (4.3–8.3)			0.350	
Grip strength improvement 12m, kg		1.2 (-2.1–3.1)			-0.60 (-1.70–5.22)			1.1 (-0.55–3.8)			0.098	
By gender:												
	Male n = 63						Female n = 71					
	-9-9		-9+9	+9+9		P	-9-9		-9+9	+9+9		P
Grip strength, kg	23.6 (19.0–27.6)		23.5 (20.3–26.9)	22.2 (16.2–25.6)		0.397	12.4 (10.7–16.8)		14.1 (11.3–16.6)	12.6 (9.9–16.7)		0.725
6MWD, m	321 (219–378)		354 (280–414)	310 (148–400)		0.327	308 (256–378)		300 (231–353)	283 (248–360)		0.779
QMVC, kg	15.2 (10.1–16.8)		14.5 (12.1–21.4)	18.0 (12.5–21.0)		0.552	9.5 (6.4–12.7)		9.0 (6.7–13.2)	10.2 (7.0–12.4)		0.901
Arm fat mass, kg	2.39 (1.33–2.62)		2.72 (2.05–3.33)	2.76 (2.33–3.41)		0.019	3.15 (2.84–3.52)		3.19 (2.31–3.83)	2.89 (2.41–4.41)		0.876
Leg fat mass, kg	6.40 (4.93–8.47)		6.48 (5.87–8.84)	7.12 (5.70–8.82)		0.582	9.53 (8.22–11.78)		9.91 (7.85–12.58)	8.39 (7.02–11.31)		0.334
Arm muscle mass, kg	5.34 (5.11–6.05)		6.19 (5.37–6.58)	5.71 (5.10–6.73)		0.117	3.39 (3.02–3.83)		3.29 (2.92–3.83)	3.42 (3.04–3.80)		0.664
Leg muscle mass, kg	15.6 (13.2–17.3)		15.8 (13.2–17.3)	15.1 (13.4–18.3)		0.634	11.1 (9.22–11.9)		10.5 (9.24–11.9)	10.6 (10.1–11.5)		0.852

6MWD six-minute walk distance; QMVC quadricep maximal voluntary contraction

There was a difference in SPPB improvement at 12 months (p<0.001); +9+9 genotype gained 1 SPPB point at 12 months, which was significantly larger change compared to -9-9 (p = 0.014), and +9–9 (p = 0.001) genotypes. To determine if perindopril treatment affected this, the same analysis was performed by treatment group—perindopril and no perindopril. Among those taking perindopril, there was no significant difference between the genotypes. However, among those taking placebo, there was a significant improvement in SPPB score in the +9+9 group (p = 0.006) and this was significantly greater than -9-9 (p = 0.029) and +9–9 groups (p = 0.012), shown in S1 Table.

### Chair-stand time

For the chair-stand test, 33 (24%) participants were unable to perform this task and thus had no time observation which prevented analysis of chair-stand time as a continuous variable. The missing values were imputed using the longest time taken by a participant to perform the chair-stand test of 68 seconds. The median of 26.5 seconds was used to dichotomise into ‘fast’ (<26.5 seconds) and ‘slow’ (≥26.5 seconds). Slow and fast groups were compared for both BDKRB2 variants. For both rs1799722 and rs5810761, there was no significant difference between the frequencies of genotypes in slow and fast groups by Chi-squared test (p = 0.560 and p = 0.987 respectively).

## Discussion

In this study we analysed the prevalence of rs1799722 and rs5810761 polymorphisms of BRDK2R and their associations with physiology measures in individuals from the LACE trial. Both alleles were in Hardy-Weinberg equilibrium with allele frequencies similar to control populations. Our study is the first to explore rs1799722 genotypes with standard measures of muscle function. In men the rs1799722 TT genotype was associated with greater 6MWD and higher muscle mass. The +9+9 rs5810761 genotype was associated with greater arm fat mass in men.

Previous studies had found athletic endurance to associate with the minor allele in the two studied variants [10–13]. Our hypothesis that the frequency of the minor allele may be lower than the general population did not transpire. This discrepancy between these studies and ours could be because our study had much older participants and it is expected environmental factors become more influential than genetic factors in the case of muscle function in older age. A meta-analysis showed that genetic influence declined with different age groups [21]. It would be conceivable that factors such as increased inactivity and chronic disease which increase with age, become increasingly influential on muscle function, weakening any benefit the variants would have provided during healthy, young age. Additionally, if the polymorphisms have other deleterious effects, people may not survive long enough to become sarcopenic, confounding any supposed advantage.

We did find that among men, the rs1799722 TT genotype group had a greater 6MWD than other genotype groups. Consistent with this, they also had higher leg muscle mass. It is not clear whether TT men have more muscle mass which allows them to walk a greater distance (6MWD), or if it is the ability to walk greater distances which increases muscle mass. These findings are also consistent with the theory that the TT genotype leads to increased BDKRB2 activity downstream through increased receptor transcription and that bradykinin is associated with enhanced muscle performance [10, 14]. Tsianos et al. showed that an excess of TT genotypes for this variant in endurance athletes compared to controls; participants from this study were predominantly male (95%) [13]. As endurance is a significant component of the 6MWD test our finding is to some extent consistent with that of Tsianos et al. However, our study and Tsianos’ included cohorts that were unalike (older and sarcopenic versus young and fit respectively) and direct comparisons may not be suitable.

We found men with the rs5810761 -9-9 genotype had lower arm fat than other genotypes, which is in keeping with the literature that this genotype has been associated with increased FFMI in COPD patients [15]. However, this did not translate to increased arm or leg muscle mass in our study. The evidence for the rs5810761 variant with respect to muscle performance is more mixed. Some studies have suggested the -9-9 genotype is associated with endurance events and performance [10, 11, 22], while others have failed to show any difference [23–29], and one actually favoured the +9+9 genotype [30]. We also found that the SPPB score improved in the +9+9 group at 12 months. This may be because of a slightly poorer baseline for this group and thus a greater potential to improve. We performed a post hoc subgroup analysis between perindopril and no perindopril groups to test if the medication may have affected this outcome and found that this persisted in the no perindopril group, but was negated by the presence of perindopril. This is a similar finding to that seen in our recent study of ACE gene variants, where perindopril negated the improvement in SPPB score seen among ID/II genotypes who were not taking perindopril [9]. The findings in the both studies may be explained by the effect of being recruited into a trial leading to increased activity and physical improvement–those taking perindopril suffered a higher rate of adverse events and poorer quality of life as reported in the trial [16], which may have reduced their motivation to remain active, and this could be most capitalised among +9+9 genotypes. Given the small sample size it is also possible that this result is a type I error.

There were no significant differences in other outcomes of muscle performance. One possible explanation may be due to distribution of skeletal muscle fibre types. Individuals with sarcopenia lose type II fibres, leading to an increased proportion of type I fibres and resembling the distribution seen in endurance athletes. Some of the outcomes measured such as grip strength and QMVC, require a larger type II fibre composition. However, unlike the ACE gene (where the ACE I allele has been linked to type I fibre type), fibre types have not been studied in BDKRB2 variants.

Another reason for the lack of association between muscle function and genotypes could be an interaction with environmental factors, namely exercise. Gacesa et al. demonstrated that the -9-9 genotype was associated with a higher increase in triceps brachii volume in response to training compared to +9-9/+9+9 combined [31]. Thus, it may be that such genotypes support response to training, and could explain its predominance in athletes. Our participants did not undergo any additional exercise or training; propensity to improve muscle function with exercise between genotypes in sarcopenia should also be studied in future.

There were limitations with our study. Our sample size is very small. The LACE trial was stopped early and underrecruited, therefore had reduced statistical power. Moreover, focussing on minor alleles (-9 and T) inevitably leads to a reduced sample size within the minor genotype subgroups. Thus, hypothesis testing of the continuous variables between the subgroups was underpowered to detect differences. Additionally, the inclusion criteria of sarcopenia results in low performing participants, which may cause floor and ceiling effects in the variables, making it difficult to discriminate between participants. Our analysed population all had sarcopenia and, as such, inferences about whether different alleles are associated with sarcopenia cannot be made, but only whether the alleles are associated with differences in strength and mass within a sarcopenic population. The study should be confirmed in a larger cohort with a matched control without sarcopenia.

## Conclusion

This study did not demonstrate a difference in the rare genotype distribution among sarcopenic individuals when compared to control groups. There is a suggestion that the genotypes may have an effect on body composition of muscle and fat in men, but not women. Further studies are required to explore this.

## Supporting information

S1 File Dataset used for analyses. (XLSX)

S1 Table Differences between genotypes in improvement in SPPB at 12 months split by perindopril. (DOCX)

## References

[1] Cruz-Jentoft AJ, Sayer AA. Sarcopenia. The Lancet. 2019 Jun;393(10191):2636–46. 10.1016/S0140-6736(19)31138-9 31171417

[2] Kemp PR, Griffiths M, Polkey MI. Muscle wasting in the presence of disease, why is it so variable? Biol Rev. 2019 Jun;94(3):1038–55. doi: 10.1111/brv.12489 30588725

[3] Powers SK, Morton AB, Hyatt H, Hinkley MJ. The Renin-Angiotensin System and Skeletal Muscle. Exerc Sport Sci Rev. 2018 Oct;46(4):205–14. doi: 10.1249/JES.0000000000000158 30001274 PMC6673677

[4] Brink M, Price SR, Chrast J, Bailey JL, Anwar A, Mitch WE, et al. Angiotensin II Induces Skeletal Muscle Wasting through Enhanced Protein Degradation and Down-Regulates Autocrine Insulin-Like Growth Factor I. 2001;142(4). doi: 10.1210/endo.142.4.8082 11250929

[5] Du Bois P, Pablo Tortola C, Lodka D, Kny M, Schmidt F, Song K, et al. Angiotensin II Induces Skeletal Muscle Atrophy by Activating TFEB-Mediated *MuRF1* Expression. Circ Res. 2015 Aug 14;117(5):424–36. 26137861 10.1161/CIRCRESAHA.114.305393 PMC4537692

[6] Rigat B, Hubert C, Alhenc-Gelas F, Cambien F, Corvol P, Soubrier F. An insertion/deletion polymorphism in the angiotensin I-converting enzyme gene accounting for half the variance of serum enzyme levels. J Clin Invest. 1990 Oct 1;86(4):1343–6. doi: 10.1172/JCI114844 1976655 PMC296868

[7] De Mello Costa MF, Slocombe R. The Use of Angiotensin-I Converting Enzyme I/D Genetic Polymorphism as a Biomarker of Athletic Performance in Humans. Biosensors. 2012 Oct 9;2(4):396–404. doi: 10.3390/bios2040396 25586030 PMC4263561

[8] Zhang B, Tanaka H, Shono N, Miura S, Kiyonaga A, Shindo M, et al. The I allele of the angiotensin‐converting enzyme gene is associated with an increased percentage of slow‐twitch type I fibers in human skeletal muscle. Clin Genet. 2003 Feb;63(2):139–44. doi: 10.1034/j.1399-0004.2003.00029.x 12630962

[9] Rossios C, Bashir T, Achison M, Adamson S, Akpan A, Aspray T, et al. ACE I/D genotype associates with strength in sarcopenic men but not with response to ACE inhibitor therapy in older adults with sarcopenia: Results from the LACE trial. Hitachi K, editor. PLOS ONE. 2023 Oct 20;18(10):e0292402. 10.1371/journal.pone.0292402 PMC10588903 37862321

[10] Williams AG, Dhamrait SS, Wootton PTE, Day SH, Hawe E, Payne JR, et al. Bradykinin receptor gene variant and human physical performance. J Appl Physiol. 2004 Mar;96(3):938–42. doi: 10.1152/japplphysiol.00865.2003 14607851

[11] Saunders CJ, de Milander L, Hew-Butler T, Xenophontos SL, Cariolou MA, Anastassiades LC, et al. Dipsogenic genes associated with weight changes during Ironman Triathlons. Hum Mol Genet. 2006 Oct 15;15(20):2980–7. doi: 10.1093/hmg/ddl240 16950802

[12] Braun A, Kammerer S, Maier E, Böhme E, Roscher AA. Polymorphisms in the gene for the human B2-bradykinin receptor. New tools in assessing a genetic risk for bradykinin-associated diseases. Immunopharmacology. 1996 Jun;33(1–3):32–5. doi: 10.1016/0162-3109(96)00079-3 8856111

[13] Tsianos GI, Evangelou E, Boot A, Carola Zillikens M, van Meurs JBJ, Uitterlinden AG, et al. Associations of polymorphisms of eight muscle- or metabolism-related genes with performance in Mount Olympus marathon runners. J Appl Physiol. 2010 Mar;108(3):567–74. doi: 10.1152/japplphysiol.00780.2009 20044476

[14] GTEx Portal [Internet]. [cited 2024 Jan 16]. Available from: https://gtexportal.org/home/snp/rs1799722

[15] Hopkinson NS, Eleftheriou KI, Payne J, Nickol AH, Hawe E, Moxham J, et al. +9/+9 Homozygosity of the bradykinin receptor gene polymorphism is associated with reduced fat-free mass in chronic obstructive pulmonary disease. Am J Clin Nutr. 2006;83(4):912–7. doi: 10.1093/ajcn/83.4.912 16600946

[16] The LACE study group, Achison M, Adamson S, Akpan A, Aspray T, Avenell A, et al. Effect of perindopril or leucine on physical performance in older people with sarcopenia: the LACE randomized controlled trial. J Cachexia Sarcopenia Muscle. 2022 Apr;13(2):858–71. doi: 10.1002/jcsm.12934 35174663 PMC8977979

[17] Band MM, Sumukadas D, Struthers AD, Avenell A, Donnan PT, Kemp PR, et al. Leucine and ACE inhibitors as therapies for sarcopenia (LACE trial): study protocol for a randomised controlled trial. Trials. 2018 Dec;19(1):6. doi: 10.1186/s13063-017-2390-9 29301558 PMC5753568

[18] Bashir T, Achison M, Adamson S, Akpan A, Aspray T, Avenell A, et al. Activin type I receptor polymorphisms and body composition in older individuals with sarcopenia—Analyses from the LACE randomised controlled trial. Attaway AH, editor. PLOS ONE. 2023 Nov 14;18(11):e0294330. doi: 10.1371/journal.pone.0294330 37963137 PMC10645316

[19] Roberts HC, Denison HJ, Martin HJ, Patel HP, Syddall H, Cooper C, et al. A review of the measurement of grip strength in clinical and epidemiological studies: towards a standardised approach. Age Ageing. 2011 Jul;40(4):423–9. doi: 10.1093/ageing/afr051 21624928

[20] pharmgkb rs1799722 [Internet]. Available from: https://www.pharmgkb.org/variant/PA166154785

[21] Zempo H, Miyamoto-Mikami E, Kikuchi N, Fuku N, Miyachi M, Murakami H. Heritability estimates of muscle strength-related phenotypes: A systematic review and meta-analysis. Scand J Med Sci Sports. 2017 Dec;27(12):1537–46. doi: 10.1111/sms.12804 27882617

[22] Varillas-Delgado D, Morencos E, Gutierrez-Hellin J, Aguilar-Navarro M, Munoz A, Mendoza Laiz N, et al. Genetic profiles to identify talents in elite endurance athletes and professional football players. PloS One. 2022;17(9):e0274880. doi: 10.1371/journal.pone.0274880 36112609 PMC9480996

[23] Varillas-Delgado D, Telleria Orriols JJ, Del Coso J. Genetic Profile in Genes Associated with Cardiorespiratory Fitness in Elite Spanish Male Endurance Athletes. Genes. 2021;12(8). doi: 10.3390/genes12081230 34440404 PMC8391315

[24] Zmijewski P, Grenda A, Leońska-Duniec A, Ahmetov I, Orysiak J, Cięszczyk P. Effect of BDKRB2 Gene −9/+9 Polymorphism on Training Improvements in Competitive Swimmers. J Strength Cond Res. 2016 Mar;30(3):665–71. doi: 10.1519/JSC.0000000000001145 26907838

[25] Sawczuk M, Timshina YI, Astratenkova IV, Maciejewska-Karłowska A, Leońska-Duniec A, Ficek K, et al. The -9 /+9 Polymorphism of the Bradykinin Receptor Beta 2 Gene and Athlete Status: A Study Involving Two European Cohorts. Hum Biol. 2013 Oct;85(5):741–55. doi: 10.3378/027.085.0511 25078958

[26] Sgourou A, Fotopoulos V, Kontos V, Patrinos GP, Papachatzopoulou A. Association of genome variations in the renin-angiotensin system with physical performance. Hum Genomics. 2012;6(101202210):24. doi: 10.1186/1479-7364-6-24 23176367 PMC3543191

[27] Eynon N, Meckel Y, Alves AJ, Nemet D, Eliakim A. Is there an interaction between BDKRB2−9/+9 and GNB3 C825T polymorphisms and elite athletic performance?: BDKRB2 and GNB3 genes and elite athletes. Scand J Med Sci Sports. 2011 Dec;21(6):e242–6. 21210858 10.1111/j.1600-0838.2010.01261.x

[28] Gronek P, Gronek J, Lulinska-Kuklik E, Spieszny M, Niewczas M, Kaczmarczyk M, et al. Polygenic Study of Endurance-Associated Genetic Markers NOS3 (Glu298Asp), BDKRB2 (-9/+9), UCP2 (Ala55Val), AMPD1 (Gln45Ter) and ACE (I/D) in Polish Male Half Marathoners. J Hum Kinet. 2018;64(101513031):87–98. 30429902 10.1515/hukin-2017-0204 PMC6231335

[29] Grenda A, Leonska-Duniec A, Cieszczyk P, Zmijewski P. Bdkrb2 gene -9/+9 polymorphism and swimming performance. Biol Sport. 2014;31(2):109–13. doi: 10.5604/20831862.1096047 24899774 PMC4042657

[30] Neto SLDA, Herrera JJB, Rosa TS, de Almeida SS, Silva GCB, Ferreira CES, et al. Interaction Between ACTN3 (R577X), ACE (I/D), and BDKRB2 (-9/+9) Polymorphisms and Endurance Phenotypes in Brazilian Long-Distance Swimmers. J Strength Cond Res. 2022;36(6):1591–5. doi: 10.1519/JSC.0000000000003685 32639377

[31] Popadic Gacesa JZ, Momcilovic M, Veselinovic I, Brodie DA, Grujic NG. Bradykinin type 2 receptor -9/-9 genotype is associated with triceps brachii muscle hypertrophy following strength training in young healthy men. BMC Musculoskelet Disord. 2012 Dec;13(1):217. doi: 10.1186/1471-2474-13-217 23127247 PMC3531309

