# Supplementary material for: Association of bradykinin receptor 2 (BDKRB2) variants with physical performance and muscle mass: Findings from the LACE sarcopenia trial
Source: PLoS One. 2024 Aug 2;19(8):e0307268. doi: 10.1371/journal.pone.0307268 (PMC11296637; doi:10.1371/journal.pone.0307268)
Supplement: S1 Table — (DOCX) [file pone.0307268.s002.docx]

| S1 table: differences between genotypes in improvement in SPPB at 12 months split by perindopril | | | | | | | | |
| --- | --- | --- | --- | --- | --- | --- | --- | --- |
|  | Perindopril | | | | No perindopril | | | |
|  | -9-9 | -9+9 | +9+9 | P value | -9-9 | -9+9 | +9+9 | P value |
| Change SPPB | -0.50 | 0 | 1.0 | 0.169 | 0 | 0 | 1.0 | 0.006 |
